# Emotional affection on a sustained attention task: The importance the aging process and depression

**DOI:** 10.1371/journal.pone.0234405

**Published:** 2020-06-29

**Authors:** Luis Pinel, Miguel A. Perez-Nieto, Marta Redondo, Luis Rodríguez-Rodríguez, Fernando Gordillo, Leticia León

**Affiliations:** 1 Department of Education and Health, Camilo José Cela University, Madrid, Spain; 2 Department of Rheumatology, Hospital Clínico Universitario San Carlos, Madrid, Spain; Victoria University of Wellington, NEW ZEALAND

## Abstract

Chronic pain is a complex experience that has now become a major public health issue. This has prompted many researchers to study attention, understanding it to be a crucial factor that allows altering the experience of pain, while attributing considerable importance to sustained attention. Accordingly, the main studies in this field stress the importance of emotion regulation processes and emotions on the perception of painful stimuli and attentional processes themselves. Nevertheless, only a handful of studies have been found that directly study the relationship between these variables. Within this context, this article sets out to analyse emotional regulation processes, emotional variables (depression and anxiety), the experience of pain, and age on the ability to maintain the vigilance response in a sample of patients with chronic pain. This involved selecting a sample of 49 patients with rheumatoid arthritis and examining their performance in an ad-hoc sustained attention test. With a view to complying with the study’s main purpose, the participants were also assessed through the use of the following self-report measures: the Beck Depression Inventory (BDI-I); the Hospital Anxiety and Depression Scale (HADS); the McGill Pain Questionnaire, and the Difficulties in Emotion Regulation Scale (DERS). Linear regression analyses revealed a significant impact of the aging process on the performance times in the attention task. Likewise, age and depression recorded a significant correlation with the mistakes made during the task. These results suggest that higher depression levels and an older age might be related to a worse adaptation to pain management techniques based on attention processes, such as mindfulness.

## 1. Introduction

Chronic pain is a complex experience that has become a major public health issue not only because of its alarmingly high rate of occurrence [1], but also because of its steady growth in recent decades [2]. Its negative impact on the quality of life of those who suffer from it, the frequent psychological after-effects, the costs incurred by the loss of productivity at work, and the high socio-economic burden on the health system [3, 4] have turned this multidimensional phenomenon into a key subject of study and interest in today’s health sciences.

Some authors now stress the importance of understanding this health issue from a biopsychosocial perspective [5], whereby physiological components [6] interact with psychological and social factors to inform the experience of pain. This means that emotional and cognitive factors have a considerable influence on the perception of painful stimuli [7]. Attention therefore emerges as a key factor, as it acts as an intermediary that allows selecting sensory events and their appearance in awareness [8]. Hence the reason that the study of attentional processes in relation to chronic pain has been an overriding priority for the scientific community [8, 9]. Today’s attention models distinguish between two types of information processing: bottom-up and top-down, which are also referred to as exogenous and endogenous attention, respectively [10–12]. Pain’s very nature makes it a stimulus that is very hard to ignore, capturing attention and automatically rerouting it [13], due to among other factors its importance for the sufferer [14]. This capturing of attention is known as bottom-up processing and is directed by stimuli, being often defined as automatic or involuntary. Once pain as been captured by attention, an individual may increase their vigilance of it and focus on nociceptive stimuli [15], with the aim being to diminish its perception, remedying or even avoiding it [16–18]. This is a voluntary top-down process guided by an individual’s goals and motivations [19, 20]. The cost of focusing one’s attention on painful stimuli not only maintains the experience of pain, but also contributes to the experiencing of negative emotions and increases disability [21, 22]. This ability to uphold the vigilance response for a long period of time is referred to as sustained attention [23, 24]. As regards cognitive behavioural interventions, there are numerous studies that have found evidence in favour of distraction [10, 25–30] as a coping strategy for drawing attention away from pain. Mindfulness-Based Interventions (MBIs) work on sustained attention as a key aspect in the emotion regulation process with these patients [31].

Despite the importance of this attentional component in the field of chronic pain, there are very few instances of empirical research that have addressed it directly [32]. Nevertheless, sundry studies have indicated that pain can impair the performance of sustained attention tasks [33–38]. Several researchers have also provided evidence of the existence of an attentional bias in sustained attention in both humans and animals with chronic pain [39, 40]. Its significance has also been studied within the field of neuropsychology, where it has been reported that tasks of sustained attention within contexts of pain activate the anterior cingulate cortex [41, 42]. This area could be involved in the perception of pain and the way it functions [43–47]. It might therefore be affirmed that sustained attention is a key factor both in the theoretical ambit and in the clinical context within the field of chronic pain. More research is therefore called for on the variables with an impact on it.

Therefore, this psychological construct “sustained attention” might have an implication in the clinical field, more accurately in the selection of the criteria for guiding a doctor’s choice of treatment. In this regard, we decided to conduct a broader analysis of the relevant literature looking for psychological variables that could interfere with the vigilance response. In general terms, the underlying mechanism by which this ability to uphold the vigilance response is affected by other variables is unclear. Nonetheless, according to posner's attentional model [48, 49] emotional factors play a crucial role in sustained attention. Posner described an attentional model based on three different brain networks: the alerting system in charge of achieving a state of receptiveness towards incoming stimuli, the orienting system that is destined to direct attention to a target stimulus, and the executive attention system for monitoring performance and carry out functions that can be described in cognitive terms. As described by the author, the executive control of attention involves mechanisms for monitoring and resolving conflict among thoughts, feelings, and responses. The fact that emotions tend to influence the cognitive response is well-established thanks to the ‘Three-Systems-Model’ [50, 51]. These evidences suggest that emotional factors can play an important role in sustained attention, which has been elaborated considerably in the literature [52, 53]. Besides that it can provide an explanation about the reasons of why variables such as anxiety or mood have been studied in depth in relation to attentional processes, drawing the following conclusions: regarding anxiety, studies using image-based experimental tasks have found that anxious patients recorded an attentional bias toward images with a threatening content [54]. Some studies suggest that these individuals find more difficult to disengage their attention from threatening content in more elaborate stages of information processing [55], as it happens when upholding the vigilance response for a long period of time. In turn, depression has been consistently linked to a deficit in attention resources [56, 57]. Image-based experimental studies also report that depression is linked to a difficulty in attentional disengagement [58], greater attentional focus on images with a greater emotional congruence [59], and longer-lasting ones [60]. Along these same lines, several researchers have confirmed that depression is related to abnormally slow information processing times in demanding attention tasks [61, 62]. From a theoretical perspective, chronic pain patients with higher levels of anxiety and depression would exhibit more difficulty in attentional disengagement, make more mistakes, and take longer to complete any vigilance task that involve conflict.

Consequently, it can be assumed that not only emotions, but one's abilities to successfully manage emotions, could also be relevant for the study of the attentional processes. Nevertheless, very few studies have considered the impact of emotion regulation mechanisms on attention or the experience of pain. In a recent review of emotion regulation in chronic pain [63], it was pointed out that maladaptive response-focused emotion regulation is related with a negative impact on well-being, functioning, depression, anxiety and stress. Furthermore, the authors point out that it seems to be an indirect bidirectional relationship between emotion regulation strategies and pain outcomes in these patients. Other types of studies tended to lead to the same conclusion, they confirm that the difficulties in emotion regulation are linked to more intense pain, greater disability, and/or heightened emotional conflict in this population [64–69]. However, despite the reports on the relationship between these variables, its operating mechanisms are still unknown, although taking account of all available evidence, one might conclude that a deficit in this ability might modify both attentional processes and the experience of pain in these patients.

As regards the socio-demographic variables, there is evidence to show that ageing leads to a decline in the cognitive function [70–73]. Many studies find that older patients with chronic pain perform worse in empirical tests involving selective and sustained attention [38, 74–76]. These data suggest that age is an important variable that helps to explain the relationship between attentional processes and the experience of pain among these patients. Therefore, and building on previous studies, it could be predicted that an older age would be associated with worse outcomes in a demanding vigilance task in every way.

Hence the reason that our aim here was to analyse the relationship between age, depression, anxiety, rating of pain and the capacity for emotion regulation, and their predictive capacity in an ad-hoc visual task involving sustained attention, given the proven relationship between these variables and the maintenance of attention according to the main theoretical models mentioned earlier [10, 22, 48, 49]. Our remit is therefore to provide information of use in clinical practice regarding the component of sustained attention, measured through the empirical task, and the aforementioned psychological variables.

## 2. Material and method

### Participants

This study involved a sample made up of a total of forty-nine patients diagnosed with rheumatoid arthritis. All the participants had to have been experiencing pain for more than six months to comply with the criteria of the Spanish Pain Society—Sociedad Española del Dolor (SED) for chronic pain, be aged over 18, and have volunteered to take part in the study. Those patients that had been diagnosed with an anxiety or mood disorder were discarded. Some of the participants were patients undergoing treatment at the Department of Rheumatology at the Hospital Clínico San Carlos in Madrid (Spain), while others were members of the Madrid Association of Patients with Rheumatoid Arthritis (AMAPAR, in its Spanish acronym).

### Ethical statement

The study was approved by the Research and Ethics Committee of CEIC Hospital Clínico San Carlos in Spain (record 15.531-E). Date of communication: 10 December 2015. Written informed consent was obtained from all participants after been informed about eligibility criteria, study procedures and study goal. Data collection regarding the consent was witnessed and supervised by the lead investigator. Only participants who completed data for all self-report measures listed below were included in the sample and taken into consideration for the statistical analyses. Those who did not meet the eligible criteria, as noted previously, were discarded and advised to seek psychological treatment.

### Assessment instruments

#### Self-report measures

- **BDI-II**. Beck’s Depression Inventory-II by Beck et al. [77], in its abbreviated form adapted into Spanish by Sanz et al. [78]. This self-report instrument consists of 11 items that, with good scores in terms of validity and reliability, use a four-point Likert-type scale to quantify the seriousness of the depressive symptomology over the two previous weeks. Our sample recorded excellent levels of reliability and internal consistency (Cronbach’s alpha = 0.856), following the criteria proposed by Prieto et al. [79].
- **HAD–A** (Anxiety Scale). The Hospital Anxiety and Depression Scale (Zigmond et al. [80], in its Spanish version by Herrero et al. [81]). As regards its two component subscales, this study selected the one of anxiety. This measure consists of seven self-administered items that assess the seriousness of the symptoms of anxiety through a four-point Likert-type scale. The measure was selected because of its ability to reliably assess the symptoms of anxiety among the patients. In our study, the measure has recorded appropriate levels of reliability (Cronbach’s alpha = 0.763) according to Prieto et al. [79].
- **McGill.** The McGill Pain Questionnaire (Melzack [82], in its Spanish version by Lázaro et al. [83]). The questionnaire consists of a list of 19 descriptors that report on how each patient rates their pain. It evaluates both quantitative and qualitative aspects of pain, such as location, quality, temporal properties, and intensity. In this study, this instrument has recorded appropriate levels of reliability and internal consistency (Cronbach’s alpha), specifically 0.738, which were suitable according to the criteria of Pietro et al. [79].
- **DERS**. Difficulties in Emotion Regulation Scale (Gratz et al. [84], in its Spanish version by Hervás et al. [85]). The test measures components of emotion regulation. It consists of a total of 36 items, with the responses being scored on a five-point Likert-type scale: from 1 (almost never) to 5 (almost always). It is divided into five subscales that are related to the difficulties in emotion regulation, and which in our study have recorded both a minimum appropriate level of reliability and internal consistency through Cronbach’s alpha (between brackets): emotional rejection (0.875), emotional confusion (0.804), emotional disengagement (0,671), everyday interference (0.848), and emotional dyscontrol (0,834).
- **Images used as emotional prompts:** the images used in this study were taken from the International Affective Picture System (IAPS; Lang et al. [86]). IAPS is a library of 1000 pictures carefully chosen to evoke a wide variety of emotional reactions and its reliability has been tested in Spanish population [87]. Thereby making it the perfect choice for the purpose of this study, which allowed us to recreate the emotional context needed to test the vigilance response. In order to make the selection of pictures for the task, a group of experts in chronic pain was consulted. The experts independently rated each picture to ensure that the most representative ones were chosen, including the selection of pictures for the target stimulus (emotionally neutral). The selection criteria involved choosing a total of 90 images (including photos of bleeding, harm, threats, injuries, pain, etc.) of a negative valence (discomfort triggered by the image) with different levels of activation or arousal (low, medium and high) (Images: 1300, 2053, 2115, 2120, 2130, 2141, 2205, 2700, 2710, 2745.2, 2750, 2799, 3000, 3005.1, 3017, 3019, 3051, 3103, 3120, 3160, 3168, 3170, 3211, 3216, 3220, 3250, 3310, 3400, 3550.1, 5940, 5971, 6000, 6021, 6022, 6212, 6242, 6243, 6250, 6312, 6370, 6415, 6520, 6550, 6561, 6563, 6570.1, 6610, 6831, 6838, 6840, 7211, 7361, 7380, 8230, 8480, 9000, 9005, 9050, 9075, 9145, 9171, 9180, 9184, 9265, 9300, 9302, 9326, 9340, 9413, 9417, 9420, 9423, 9428, 9429, 9432, 9470, 9480, 9500, 9520, 9596, 9600, 9611, 9623, 9635.1, 9800, 9901, 9903, 9905, 9920, 9926, and 9940). Also included was an image with a neutral stimulus (a photo of a wall-clock against a white background) (Image: 7211), this image was the “target” stimulus in the sustained attention task. The images were not airbrushed in any way.

#### Sustained attention task

**Fig 1:**
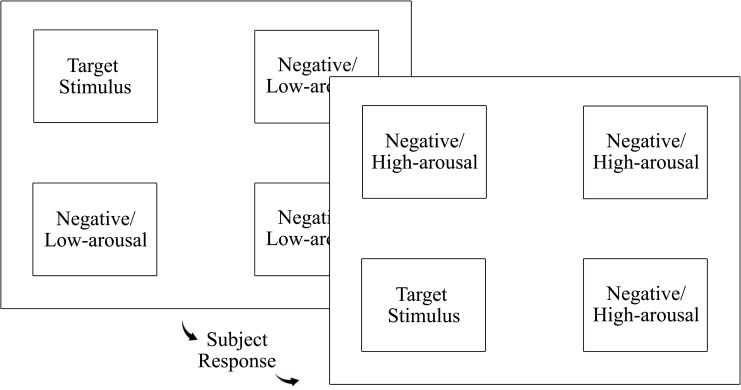
First images displayed in the sustained attention task.

Sustained attention requires an observer to maintain engagement in a task (e.g. reacting to a target stimulus) over a prolonged time interval [88]. The ad hoc task used for this study is a modified version of the Continuous performance task (CPT) paradigms with the aim of assessing sustained attention over an extended period of time. Since it has been established that the extended duration of the activity is particularly important to differentiate sustained attention from other classes of attention [23, 24], an ad hoc task was required for achieving research’s main objectives. The ad-hoc test involved displaying four images, with each one appearing in one of the four corners of the screen; three related to pain and the target stimulus (emotionally neutral image). As mentioned, all the photos were taken from the IAPS (Lang et al. [86]). The display of the stimuli was controlled by the E-run software program from version 3.0 of the E-Prime package (Psychology Software Tools, Sharpsburg, USA). The test consisted of a total of 17 series, with each one comprising 30 slides with four images each. The slides in each series were randomly displayed for each subject. A black screen was used to display the four images on each slide, one in the upper left-hand corner, one in the lower left-hand corner, another in the upper right-hand corner, and finally, one in the lower right-hand corner (see Fig 1). Each subject had to follow the target stimulus. Once the subject had made their choice, pressing the keys “v” (lower left-hand), “f” (upper left-hand), “j” (upper right-hand) and “n” (lower right-hand), the set of images was replaced by the next slide until all the series had been completed. It is assumed that those participants whose psychological variables so determined show a preference for pain-related stimuli, and therefore increase both their number of errors and reaction times when undertaking the attention test.

### Procedure

The sample’s participants were assessed in the Department of Rheumatology at the Hospital Clínico San Carlos in Madrid and at AMAPAR. The assessment was conducted in a single one-hour session by the same evaluator on a one-to-one basis. The participants began by signing an informed consent form and were told about the discomfort or the emotions they might experience when seeing the images. They were then given their instructions for beginning the task: “Four images will now be displayed. Please indicate the clock’s position” and told which keys to press: “To do so, press the following keys depending on its position on the screen: v if it is in the lower left-hand corner, n if it is in the lower right-hand corner, f if it is in the upper left-hand corner, and j if it is in the upper right-hand corner. If you have understood these instructions, please press the SPACEBAR to begin”. The next step involved a trial run with 20 neutral images. This was immediately followed by the start of the sustained attention test involving 510 slides, each one with four images (three for prompting an emotional response and the target one), as already noted. Once the attention task had been completed, the evaluator began the assessment session in which the subjects completed the self-report measures.

### Data analysis

The data were coded and analysed with version 25.0 of the SPSS statistical package. The study’s objectives were achieved by means of a stepwise multiple linear regression analysis. The independent or predictor variables were the emotional variables (depression and anxiety), DERS subscales, the scales of the pain rating index, age, and relevant sociodemographic to control their effect on pain, attentional processes or emotions, included as control variables (sex, time elapsed since the first medical diagnosis, socioeconomic status and current pharmacologic treatment of pain). we tested the contribution of variables mentioned above to the explanation of variance for each one of the dependent variables, the number of mistakes made, and the overall performance time measured in milliseconds, as computed with the attention task. Before beginning the analysis, we ensured compliance with the conditions of normality, homoscedasticity, linearity, collinearity, as required for this type of analysis. The independence was fulfilled in the first set of regression analyses (Durbin-Watson = 2.390), while the data referring to the dependent variable on the mistakes made in our study’s test revealed that they did not fulfil this condition, as the Durbin-Watson statistic (1.264) did not fall within the recommended range (2 ± 0.5). The decision was nonetheless made to conduct the analyses for this dependent variable because it met all the other conditions required. http://dx.doi.org/10.17504/protocols.io.bfcgjitw[PROTOCOL DOI]

## 3. Results

### Socio-demographic characteristics of the study participants

The mean age of study participants was 55 years (*SD* ± 10.4), ranging from 28 to 77 years. The majority of the participants were female (75.5%). All 49 patients had been diagnosed with rheumatoid arthritis and the majority were taking a combination of different medical treatments for the pain (n = 44, 89.8%), such as biological agents, FAMES, corticosteroids, anti-inflammatory or analgesic drugs. More than half were married (*n* = 25, 51%), around a quarter were single (*n* = 13, 26.5%), the rest were widowed (*n* = 3, 6.1%), divorced (*n* = 4, 8.2%) or separated (*n* = 4, 8.2%). The majority of the participants were found to be employed (*n* = 21, 42.8%), nearly one-quarter were retired (*n* = 12, 24.5%), and the rest were students (*n* = 2, 4.1%) or had other employment status (*n* = 14, 26.5%). Many individuals reported medium incomes (*n* = 32, 65.3%). Regarding their educational status, most of participants were attending primary or secondary school (*n* = 25, 51%), another important part of participants was enrolled in some type of tertiary or vocational education (*n* = 22, 44.9%). As regards the time elapsed since the first medical diagnosis, (n = 41, 83.7%) of study participants had been diagnosed with rheumatoid arthritis between five and ten years or more than ten. and around one-third was single (*n* = 120, 34.2%). The characteristics of the study participants, based on socio-demographics and relevant clinical variables are summarized in Table 1.

**Table 1:** Sociodemographic characteristics and clinical variables of the study participants.

Characteristic	Frequency (n)	Percentage (%)
**Sex**		
Female	37	75.5
Male	12	24.5
**Age in years** *(M*, *SD)*	55.2 ± (10.4)	
**Marital status**		
Single	13	26.5
Married	25	51.0
Widowed	3	6.1
Divorced	4	8.2
Separated	4	8.2
**Education level**		
Primary	6	12.2
EGB or equivalent	7	14.3
Technical and Vocational	10	20.4
Senior high school	12	24.5
University	10	20.4
Higher education	2	4.1
Unregulated studies	2	4.1
**Employment status**		
Service Sector	8	16.3
Administrative services jobs	7	14.3
Professional or Technician	5	10.2
Housewife	2	4.1
Student	2	4.1
Retired	12	24.5
Armed Forces Professionals	1	2
Unemployed	6	10.2
Others	6	12.2
**Socioeconomic status**		
Low	12	24.5
Medium	32	65.3
High	5	10.2
**Diagnosis**		
Rheumatoid arthritis	49	100
**Time elapsed since the first medical diagnosis**		
Less than a year	2	4.1
Less than three years	4	8.2
Less than five years	2	4.1
Between five and ten years	14	28.6
More than 10 years	27	55.1
**Pharmacological Treatment**		
Biological agents (e.g. Infliximab, Abatacept, etc.).	2	4.1
FAMES (e.g. Metotrexato).	1	2
Corticosteroids	0	0
Anti-inflammatory drugs	0	0
Analgesic drugs	0	0
Others	2	4.1
Several of the above	44	89.8

M = Mean; SD = Standard deviation.

### Multiple regression analyses of times and mistakes made in the attention task

The first analysis involved taking the dependent variable to be the total time the participants needed to complete the empirical task (measured in milliseconds), with the independent variables listed above. The regression analyses conducted produced a statistically significant model (F = 26.303, p ≤ 0.01) explaining around 34% of the variability in the data regarding total performance times in the attention task in our sample (Adjusted R^2^ = 0.345), including the patients’ age as sole predictor (*Beta* = 0.599, p ≤ 0.01) (Table 2). The linear regression analyses reveal a positive and significant correlation between age and the dependent variable, recording a moderate effect size in relation to Cohen’s criteria [89].

**Table 2:** Stepwise multiple linear regression analysis of difficulties in emotion regulation, depression, anxiety, age, the pain index scales and control variables on total performance times in the attention task.

**Step**	Predictors						Regression model			
		*B*	β	t	p	R	R²	ΔR²	F	p
1	Constant	176187.187		4.784	0.01					
	Age	3362.822	0.599***	5.129	0.01	0.35	0.34	0.359	26.303***	0.01

N = 49. * p < 0.05; ** p < 0.01; *** p < 0.001. B = Non-standardised regression coefficient. Beta = Standardised regression coefficient. Age: participants’ age in years.

In a next set of regression analyses we tested the contribution of variables mentioned above to the explanation of variance in number of mistakes made in the sustained attention task. In the first step, the analyses revealed a model statistically significant (F = 5.399, p = 0.025) and it explained a total of 8.4% with age as unique predictor. With the addition of depression (ΔR² = 0.103) in the second step, the model increased its predictive power accounting for up to 17% of the explained variance (F-change = 6.009, p ≤ 0.18), leading to a statistically significant improvement (F = 5.991, p ≤ 0.05) in its capacity for predicting the dependent variable. Thus, the final model revealed by the analysis included two variables as a predictor of the participant’s number of mistakes made in the attention task, age and depression (Table 3), obtaining a “small” effect size according to the criteria proposed by Cohen [89].

**Table 3:** Stepwise multiple linear regression analysis of difficulties in emotion regulation, depression, anxiety, age and the pain index scales on mistakes made in the attention task.

**Step**	Predictors						Regression model			
		*B*	β	t	p	R	R²	ΔR²	F	p
1	Constant	-6.558		-0.834	0.40					
	Age	0.325	0.321*	2.323	0.02	0.10	0.84	0.103	5.399*	0.025
2	Constant	-14.616		-1.791	0.08					
	Age	0.413	0.407*	2.996	0.004	0.20	0.17	0.104	5.991**	0.005
	BDI	0.775	0.333**	2.456	0.018					

N = 49. * p < 0.05; ** p < 0.01; *** p < 0.001. B = Non-standardised regression coefficient. Beta = Standardised regression coefficient. Age: participants’ age in years. BDI: Abbreviated version of the Beck Depression Inventory. PRI Total: Pain Rating Index scales measured by the McGill Pain Questionnaire.

## 4. Discussion

The present study investigated the relationship between psychological variables and attentional performance in chronic pain patients. To do this, a vigilance task made in an ad-hoc manner has been used to meet the proposed objectives. Results concerning the relationship between age and attentional performance show that age is a predictor variable of both the time the participants took to complete the task and the number of mistakes made. The relationship was direct and statistically significant in both cases, which means ageing is accompanied by an increase in the time required for completing the task and by more mistakes. Based on the literature reviewed, cognitive approach studies provided evidence that pain competes for limited attentional resources [90–92], and many previous studies have related increasing age with decline in attentional functions in general population [70, 73, 93]. Our results partially contributed to support this evidence that pain competes with the available attentional resources, although we used a sample of chronic pain patients. Moreover, some studies revealed a direct connection between older pain patients and poor performance on selective and sustained attention tasks [74–76]. The findings are fully consistent with all of these studies suggesting that age is an important variable that helps to explain the relationship between attentional processes and the experience of pain among these patients and it seems to be is an important variable to take into account when selecting the psychological treatment most likely to be successful.

As regards the relationship between depression and the subject’s performance in the attention test, the results reveal a significant relationship with the mistakes made during the empirical task. The attention bias commonly appears in depression and, within this context, prompts the subject to focus their attention on the stimuli most closely related to a negative emotional tone [59]. A possible explanation for these results is that attention tends to focus more on emotionally consistent images than on mood, which hinders the monitoring of the target stimulus and more mistakes are made, which is line with the findings reported in other studies [59, 60]. Furthermore, these results might imply that these patients find emotional disengagement more difficult within this context, a finding that has already been reported by other researchers [58]. In terms of performance speed, this study has been unable to find a relationship between depression and an increase in the time required to complete the empirical task, even though other scholars have indicated that sadness decreases the subject’s information processing speed [61, 62, 94]. Thus, the results for this variable are in line with what we had been hypothesized based on the analysis of the literature beforehand.

It was hypothesized that patients suffering anxiety should have performed worse in an attention performance task of this kind, our results have not found a relationship between this emotion variable and the results in the sustained attention task. As mentioned, other researchers have already contended that anxiety is associated with a greater hypervigilance of painful stimuli, a deficit in attention resources, or more difficulty in disengaging one’s attention in the presence of pain [54, 58, 95]. More research is therefore called for on this topic to understand the role that anxiety plays in these processes.

Regarding the expression of pain (using the McGill questionnaire), we haven't found that this variable correlates with the mistakes made. At least in theory, we expected some sort of relationship between the rating of pain and the mistakes made in the task. On the one hand, some studies have shown an impairment in the performance of sustained attention tasks in the presence of pain [33–38]. This was also to be expected given the nature of the experiment. On the other hand, one might argue that a long and repeated exposure to pain might lead to its normalisation, which, in turn, has been related to more automatic responses associated with it and to a lower level of interference [96–99]. In this context and with this in mind, these individuals could have made fewer mistakes and have more means for identifying the vigilance response toward a neutral stimulation, and greater ease in upholding it. But it has not been possible to confirm that either. In any case, we believe that further studies are needed to learn more about the relationship between pain and sustained attention performance during a task like the one used in the present study.

The results related to difficulties in emotion regulation, which were measured through the subscales of DERS, appear to be statistically irrelevant to predict the attentional performance. Although the role of emotion regulation and its relationship with attentional processes have been reported and some researchers suggested the importance of the difficulties in emotion regulation among patients with chronic pain [64–69], these findings do not appear to be supported by our data. Hence we could not find that difficulties in emotion regulation are associated with an interference in executive attention, where sustained attention can be included. Further research is needed to determine whether a poor emotional control is a relevant factor to guide clinical decisions in a consultation setting.

In the light of the above findings, the results suggest that patients with depression or older age could face difficulties or impairment in sustained attention and, therefore, might be related to a worse adaptation to pain management techniques based on these attention processes, such as Mindfulness-Based Interventions (MBIs) [31]. Particularly in the last decade, MBIs have been used with chronic pain patients for promoting a better emotional performance, reduce hypervigilance, and consolidate a functional improvement [31, 100–102], nevertheless, there are only few studies on this subject that examines its effectiveness taking into consideration the above-mentioned variables. It would be advisable that future researchers could take advantage of this knowledge to maximise the effectiveness of the technique.

There is another relevant issue to mention in connection with the fact that the sustained attention task was specifically designed for this study. To these it adds that there are very few studies that focus on sustained attention in the pain field. Accordingly, it is difficult to compare the findings in this study with the findings provided by other researchers. Future researchers should note issues such as the display times of images to patients with anxiety, the use of different self-report measures to evaluate emotion regulation processes or pain measures, or even the attentional task itself. These variables may change the results obtained in this study. Account must, therefore, be taken of these particular issues if we are to achieve more comprehensive results in future studies.

Over the course of this research, we have encountered certain limitations or difficulties that need to be discussed. The sample of patients with chronic pain we have accessed (n = 49) is relatively small, restricting both the treatment of the data and the statistical analyses that have been conducted. Other researchers that have already studied sustained attention in relation to chronic pain have used samples of a similar size [37–39, 103–107]. Nevertheless, our view is that a larger sample would have increased the reliability of the results obtained. Furthermore, no control was made of certain variables, such as motivation, which we know can interfere with the performance in attention tasks among this population [108]. A further experimental limitation involves the choice of the images used to prompt the emotional response. Although an empirically validated set of images has been used to evoke emotions in Spanish population [87] and create the emotional context needed for the experimental task with the objectives previously explained, this study has not controlled the possible predictive validity these images may have regarding the emotion variables in this kind of sample due to the ad hoc nature of this task. Moreover, selection of the images focuses solely on the feedback provided by the group of experts consulted on the matter, it would have been advisable to use a control task, for example, identifying the clock presented with other neutral images, with the goal of ensuring conceptual and methodological quality of the study. Finally, it should be stressed that these limitations are simply working guidelines that should be considered with a view to future studies seeking to address this matter.

In sum, this study helps to indicate the importance of emotion variables and their ability to modify attentional processes, although scholars have traditionally adopted the opposite approach. Despite its limitations, the study’s results suggest that variables such as age and depression have a negative impact on their ability to maintain the response of attentional vigilance, and indirectly provide evidence on the variables involved in the difficulty in disengaging from pain-related stimuli. In turn, the scarcity of studies on sustained attention fosters the need for its further understanding, which will enable us to discover whether there are more variables that may have an impact on its proper functioning. In the event that this information should exist, it could help to improve the interventions undertaken with these patients within the field of healthcare.

## Supporting information

S1 Data (ZIP)
